# Are all immigrant mothers really at risk of low birth weight and perinatal mortality? The crucial role of socio-economic status

**DOI:** 10.1186/s12884-016-0860-9

**Published:** 2016-04-08

**Authors:** Judith Racape, Claudia Schoenborn, Mouctar Sow, Sophie Alexander, Myriam De Spiegelaere

**Affiliations:** Research centre in Epidemiology, Biostatistics and Clinical research, Ecole de Santé Publique, Université Libre de Bruxelles(ULB), CP598. Route de Lennik 808, Bruxelles, 1070 Belgium; Research centre in Health Policies and Health Systems, Ecole de Santé Publique, Université Libre de Bruxelles (ULB), Bruxelles, 1070 Belgium; Ecole de Santé Publique, Université de Montréal, Montréal, H3N 1X9 Québec Canada

**Keywords:** Perinatal health, Low birth weight, Perinatal mortality, Health inequalities, Immigrants, Socioeconomic status

## Abstract

**Background:**

Increasing studies show that immigrants have different perinatal health outcomes compared to native women. Nevertheless, we lack a systematic examination of the combined effects of immigrant status and socioeconomic factors on perinatal outcomes. Our objectives were to analyse national Belgian data to determine 1) whether socioeconomic status (SES) modifies the association between maternal nationality and perinatal outcomes (low birth weight and perinatal mortality); 2) the effect of adopting the Belgian nationality on the association between maternal foreign nationality and perinatal outcomes.

**Methods:**

This study is a population-based study using the data from linked birth and death certificates from the Belgian civil registration system. Data are related to all singleton births to mothers living in Belgium between 1998 and 2010. Perinatal mortality and low birth weight (LBW) were estimated by SES (maternal education and parental employment status) and by maternal nationality (at her own birth and at her child’s birth). We used logistic regression to estimate the odds ratios for the associations between nationality and perinatal outcomes after adjusting for and stratifying by SES.

**Results:**

The present study includes, for the first time, all births in Belgium; that is 1,363,621 singleton births between 1998 and 2010. Compared to Belgians, we observed an increased risk of perinatal mortality in all migrant groups (*p* < 0.0001), despite lower rates of LBW in some nationalities. Immigrant mothers with the Belgian nationality had similar rates of perinatal mortality to women of Belgian origin and maintained their protection against LBW (*p* < 0.0001). After adjustment, the excess risk of perinatal mortality among immigrant groups was mostly explained by maternal education; whereas for sub-Saharan African mothers, mortality was mainly affected by parental employment status. After stratification by SES, we have uncovered a significant protective effect of immigration against LBW and perinatal mortality for women with low SES but not for high SES.

**Conclusions:**

Our results show a protective effect of migration in relation to perinatal mortality and LBW among women of low SES. Hence, the study underlines the importance of taking into account socioeconomic status in order to understand more fully the relationship between migration and perinatal outcomes. Further studies are needed to analyse more finely the impact of socio-economic characteristics on perinatal outcomes.

## Background

Health inequalities are present during pregnancy and around the time of birth for mothers and newborns, with disparities observed in maternal health, perinatal mortality, and birth outcomes [1]. Migrants seem to be particularly at risk of adverse outcomes, although studies from different high-income countries have shown conflicting results [2–5]. The perinatal health of migrants has sometimes been found to be better and other times worse than host country mothers. The divergence in results could be explained by the fact that studies differed in maternal country of origin, receiving country, and the specific outcomes (LBW, preterm birth or perinatal mortality). These mixed results also depend on the study design, on the adjustment variables and on the way that socioeconomic factors were integrated in the studies.

A large amount of research in the US Latino population has uncovered the ‘epidemiological’ or ‘immigrant paradox’, whereby immigrant women are shown to benefit from better pregnancy outcomes than native women despite their lower socioeconomic status [6–8]. Yet, a number of studies have found that the strength and direction of the association between immigration and LBW can vary according to maternal birthplace and SES [7, 9, 10]. For instance, Acevedo-Garcia et al., have shown that the protective effect of LBW varies considerably by maternal education across racial/ethnic groups [9]. It is also important to note that most studies devoted to the association between SES and perinatal health in migrants have focused on birth outcomes such as birth weight and pre-term birth, but few have studied perinatal mortality. In addition, socioeconomic information has generally been limited to maternal educational level [11].

We have shown in our previous research in Brussels a clear pattern of perinatal health inequalities among immigrants, but also some paradoxical results [5, 12–14]: Despite having favourable birth outcomes in terms of LBW and preterm birth, babies born to North African mothers had a higher risk of perinatal mortality compared to Belgians. Those differences did not persist after adopting the Belgian nationality [12]. Socio-economic factors also played an important role in determining perinatal mortality, although their impact differed by nationality [13]. In our previous study we were limited by missing values for maternal education and by small samples for some nationalities. Drawing on complete national data, this study will be able to overcome these limitations.

The aim of our study was to describe perinatal health according to the mother’s nationality and to investigate whether this association varies by SES. Hence, the objectives of our work are the following: 1) To evaluate the role of socioeconomic status (parental employment status and maternal education) as a potential confounder or modifying factor of the association between maternal nationality on one hand, and LBW and perinatal mortality on the other; 2) To assess the influence of maternal nationality (at her own birth and at her child’s birth) on perinatal mortality and LBW.

## Methods

### Study population and data

We used data from linked birth and death certificates taken from the Belgian civil registration system. The data relates to all singleton babies born between 1st January 1998 and 31st December 2010, whose mothers were living in Belgium, including those of asylum seekers and undocumented residents. In Belgium, all live births and foetal deaths from 22 completed weeks of gestation or a birth weight >500 g must be registered. Medical data are registered in hospital by midwives and gynecologists, and socio-economic data are recorded at the civil registration service within 15 days of the birth as reported by the parent(s). In Belgium, two perinatal epidemiological centers, SPE for the Dutch-speaking region and CEpiP for the French-speaking region are responsible for the quality and the completeness of the data encoded in birth and death certificates. They collaborate with the maternity hospitals and civil registration services in order to improve the quality of the data and avoid missing data.[15].

The data collection was carried out by Statistics Belgium and was exempted by law from requiring ethical approval. The process of obtaining data from linked death and birth certificates from the Belgian civil registration system is regulated by the Belgian Commission for the Protection of Privacy. After approval of this Commission, Statistics Belgium provided the data for the period 01/01/1998 to 31/12/2010.

### Definitions of the exposures and outcomes

From the birth certificates we extracted the following data: gestational age at birth, birth weight, maternal age, parity, maternal nationality of origin and at delivery, maternal educational level, maternal and paternal employment status, and if the mother is living alone or not.

We computed perinatal mortality, defined as foetal deaths from 22 weeks of gestation until 7 days after birth. We considered a birth weight to be low (LBW) when below 2,500 g.

Maternal nationality is recorded as declared at the civil registration service. We defined nationality of origin as the nationality at birth and the current nationality as the nationality at the child’s birth. We classified nationalities into the six most represented groups in the country, namely Belgium, members of the European Union (EU 27), Turkey, Maghreb, sub-Saharan Africa, Eastern European countries not included in the EU 27 and Russia. In this article, we have not shown the nationality category “others” because of the small number of subjects it contains and their heterogeneity (2.3 %). We defined naturalisation as the adoption of the Belgian nationality by the mother before delivery. Finally, we created eleven categories of nationalities, which took into account the mother’s nationality of origin and whether she subsequently acquired the Belgian nationality.

The maternal level of instruction was organised into five categories: superior (university or higher education), upper secondary (completed secondary school), lower secondary (up to the third completed year of secondary school), lower primary (completed primary or less), and other. Data on paternal and maternal occupational status were combined to derive the number of parents employed (two, one, or none). For single mothers, the paternal status was not taken into account, so in this case the number of parents employed could only vary between zero and one. We defined the number of parents employed as “parental employment status”.

Parity was categorised into three groups (nulliparity, 1 or 2 births, and > 3 births). Age of the mother was also categorised into three groups (<20 years, 20 to 40 years, and ≥ 40 years).

### Statistical analysis

We used logistic regression to estimate odds ratios (ORs) for the associations between maternal nationality and perinatal outcomes (LBW and perinatal mortality), adjusted for maternal age, parity, education and employment status. First, the crude associations between nationality and perinatal outcomes were estimated. Thus, a series of multivariate models were developed to assess the individual effects of education and employment status as potential confounding variables..In the first multivariable model we examined the influence of parity and maternal age only (Model 1); in Model 2 we added the parental employment status; in Model 3 we adjusted for parity, maternal age and maternal education; and finally, in Model 4, we included all variables, namely parity, maternal age, parental employment status and maternal education. In order to study the interaction of SES with the association between nationality and perinatal outcomes, we stratified our models by parental employment status and maternal education. We present the odds ratios derived from the logistic regressions and the p-values for the Wald *χ*^2^ test. The significance level was set at α = 0.05, and all of the analyses were performed using Stata 12 software.

## Results

### Maternal characteristics

Table 1 shows the distribution of births according to maternal nationality and socio-demographic characteristics. 1,363,621 singleton births between 1998 and 2008 were included in our study. Seventy-five percent of births were to Belgian mothers, with the other most represented subgroups being EU27 (7.2 %) and Maghreb (3.8 %). Forty per cent of sub-Saharan African mothers lived alone and in 58 % of their households none of the parents were employed. The highest proportion of young mothers (<20 years) was found among Turks and Eastern Europeans (7.6 % and 8.1 % respectively). Belgian mothers had the highest proportion of superior educational level (42.2 %), whereas Turkish, Maghrebi and Eastern European mothers were most likely to have a less than primary educational level (26.4 %, 23.8 % and 23.4 % respectively). The proportion of women in the category “other educational level” was significant for Eastern Europe (25.2 %), sub-Saharan Africa (17 %) and Turkey (17.8 %). Belgian mothers had the highest proportion of both parents being employed in the household (71.4 %), contrasting with Eastern European mothers who had the highest proportion of none of the parents being employed (61.4 %). Naturalized mothers had a higher level of education and proportion of both parents being employed in the household compared to non-naturalized mothers.

**Table 1:** Distribution of all singleton births according to maternal sociodemographic characteristics

*N* = 1 363 621*	Belgium (*n* = 1 029 471)	EU27 (*n* = 98 189)	EU27 naturalized Belgian (*n* = 34701)	Turkey (*n* = 20 451)	Turkey naturalized Belgian (*n* = 21 878)	Maghreb (*n* = 51 224)	Maghreb naturalized Belgian (*n* = 46 681)	Sub-Saharan Africa (*n* = 26 621)	sub Saharan Africa naturalized Belgian (*n* = 11 420)	East Europe (*n* = 17 420)	East Europe naturalized Belgian (*n* = 5 412)
% of births	75.5	7.2	2.5	1.5	1.5	3.8	3.4	2.0	0.85	1.3	0.4
Parity (n)	1 017 930	94 096	34 351	19 824	21 519	49 174	45 687	25 543	11 314	16 921	5 336
Nulliparity (%)	43.3	41.9	48.1	38	31.55	37.9	28.7	38.7	30.3	34.35	34.4
> = 3 children (%)	2.4	2.9	2.3	4.5	4.5	8.8	9.5	6.5	8.1	7.6	5.4
Living alone	1 016 979	96 441	34 389	20 217	21 666	50 531	46 155	26 217	11 440	17 080	5 329
Yes (%)	11.4	13.8	15.5	6.4	7.3	7.1	8.6	40.6	27.4	20.35	10.6
Maternal age (n)	1 029 446	98 181	34 699	20 449	21 878	51 220	46 679	26 615	11 572	17 412	5 412
<20 (%)	2.6	2.5	1.9	7.6	3.8	3.5	1.75	4.35	2.5	8.1	4.1
≥40 (%)	1.7	3.8	2.8	1.3	1.4	4.2	4.4	2.8	5.7	1.8	2.5
Maternal education (n)	998 773	94 957	33 138	19 544	21 216	49 663	45 604	25 829	11 305	16 825	5 308
< primary (%)	1.5	4.9	2.3	26.35	14.1	23.8	13.7	17.5	10.3	23.4	13.0
secondary inferior (%)	15.3	17.5	16.3	21.1	25.4	21.3	24.5	19.05	22.2	16.9	21.8
secondary superior (%)	35.7	35.1	36.9	30.3	41.75	31	34.1	30.15	29.3	21.1	28
superior (%)	42.2	33	36.6	5.25	7.5	8.3	14.3	15.6	26.5	13.4	24.7
other	5.4	9.5	8.0	17	11.2	15.6	13.4	17.8	11.8	25.2	12.5
Number of parents employed # (n)	983 790	90 290	33 031	19 107	20 775	47 377	44 009	23 330	10 748	14 756	5 021
0 (%)	9.5	16	12.5	34.2	26.4	33.3	23.6	58.1	31.4	61.4	24.3
1 (%)	19.2	34.7	27.5	55.1	49.7	56.8	50.3	32.8	37.7	29.6	43.5
2 (%)	71.35	49.3	60.1	10.7	23.9	9.9	26.1	9.15	30.95	9.0	32.2

*9.9 % other nationality or missing

#5.2 % missing

### Association between immigration and perinatal outcomes

Table 2 shows the crude and adjusted associations between maternal nationality and perinatal outcomes (LBW and perinatal mortality). We observed distinct patterns for LBW and perinatal mortality. In terms of LBW (Table 2), except for mothers from sub-Saharan Africa and EU27 countries, all nationalities were protected compared to Belgians. Sub-Saharan African mothers had a significantly higher risk of LBW compared to Belgians. Adjusting for maternal age and parity didn’t change the results. However, adjustment for maternal education significantly decreased the ORs of LBW in all nationality groups, and, for sub-Saharan African and EU27 naturalized women, the risks became comparable to Belgians. Adjusting for the employment status also decreased the ORs in all nationality groups, conferring protection against LBW when compared with Belgians. The ORs of for immigrant mothers were similar to those who adopted the Belgian nationality.

**Table 2:** Low birth weight (Odds ratios and crude rates) according to maternal nationality

	Per 100 live births	cases = 71,413 *n* = 1,346,789	cases = 70,460 *n* = 1,330,902	cases = 65,848 *n* = 1,263,982	cases = 67,416 *n* = 1,289,806	cases = 63,224 *n* = 1,227,848
	Crude rates	OR (95 % CI)	aOR^a^ (95 % CI)	aOR^b^ (95 % CI)	aOR^c^ (95 % CI)	aOR^d^ (95 % CI)
Belgium	5,4	1	1	1	1	1
EU27	5,3	0.98 (0.95-1.00)	0.98 (0.95-1.01)	0.84*** (0.81-0.87)	0.90*** (0.87-0.92)	0.82*** (0.79-0.84)
EU 27 naturalized Belgian	5,55	1.03 (0.98-1.07)	1.02 (0.97-1.07)	0.94* (0.89-0.98)	0.98 (0.93-1.02)	0.93** (0.89-0.98)
Turkey	4,7	0.86*** (0.80-0.92)	0.84*** (0.79-0.90)	0.64*** (0.60-0.69)	0.67*** (0.63-0.72)	0.59*** (0.55-0.64)
Turkey naturalized Belgian	4,85	0.89*** (0.83-0.95)	0.90** (0.84-0.96)	0.73*** (0.68-0.78)	0.75*** (0.71-0.80)	0.70*** (0.64-0.73)
Maghreb	3,8	0.70*** (0.67-0.73)	0.67*** (0.64-0.04)	0.49*** (0.46-0.51)	0.54*** (0.51-0.56)	0.45*** (0.43-0.47)
Maghreb naturalized Belgian	3,8	0.70*** (0.66-0.73)	0.70*** (0.66-0.73)	0.56*** (0.53-0.59)	0.59*** (0.56-0.62)	0.53*** (0.50-0.56)
Sub-Saharan Africa	6,6	1.23*** (1.17-1.29)	1.20*** (1.14-1.26)	0.78*** (0.74-0.83)	1.0 (0.95-1.05)	0.77*** (0.72-0.81)
Sub-Saharan Africa naturalized Belgian	5,9	1.10* (1.01-1.19)	1.11** (1.03-1.20)	0.85*** (0.78-0.92)	0.99 (0.91-1.07)	0.83*** (0.76-0.90)
Eastern Europe	4,9	0.90** (0.84-0.96)	0.89*** (0.83-0.95)	0.57*** (0.53-0.62)	0.72*** (0.67-0.77)	0.55*** (0.51-0.60)
Eastern Europe naturalized Belgian	4,3	0.78*** (0.69-0.89)	0.80*** (0.70-0.91)	0.66*** (0.57-0.76)	0.69*** (0.61-0.80)	0.64*** (0.56-0.73)

* ≤ 0.05 ** ≤ 0.01*** ≤ 0.001

^a^adjusted for maternal age and parity

^b^adjusted for maternal age, parity and parental employment status

^c^adjusted for maternal age, parity and maternal education

^d^adjusted for maternal age, parity, parental employment status and maternal education

In terms of perinatal mortality (Table 3), all migrant women had a significantly higher risk compared to Belgian mothers, with the exception of naturalised mothers originally from Turkey, Eastern Europe and EU 27. As with LBW, adjusting for maternal age and parity did not change the results. Adjustment for the employment status decreased the ORs to a certain extent, although mortality remained higher for migrant women (with the exception of Eastern Europe). Adjusting for maternal education significantly reduced the mortality risks for all the nationality groups, although sub-Saharan African women remained more at risk than Belgians. Finally, in the full model, only mothers from Maghreb had a slight excess mortality rate compared to Belgians albeit at the margins of significance. In contrast to LBW, immigrant mothers had higher risks of perinatal mortality than immigrant mothers who adopted the Belgian nationality.

**Table 3:** Perinatal mortality (Odds Ratios and crude rates) according to maternal nationality

	Per 1000 births	cases = 8,225 * n* = 1,363,621	cases = 8,158 * n* = 1,341,639	cases = 6,418 *n* = 1,272,917	cases = 8,069 * n* = 1,300,321	cases = 6,378 * n* = 1,236,620
	Crude rates	OR (95 % CI)	aOR^a^ (95 % CI)	aOR^b^ (95 % CI)	aOR^c^ (95 % CI)	aOR^d^ (95 % CI)
Belgium	5,6	1	1	1	1	1
EU27	6,9	1.25*** (1.15-1.35)	1.28*** (1.18-1.39)	1.12* (1.02-1.23)	1.06 (0.98-1.15)	1.02 (0.93-1.12)
EU 27 naturalized Belgian	4,2	0.76*** (0.64-0.89)	0.76*** (0.64-0.90)	0.76** (0.64-0.91)	0.69*** (0.58-0.81)	0.73*** (0.61-0.87)
Turkey	8,7	1.57*** (1.35-1.82)	1.55*** (1.34-1.81)	1.37*** (1.16-1.61)	0.99 (0.85-1.16)	1.06 (0.89-1.25)
Turkey naturalized Belgian	5,4	0.98 (0.82-1.17)	0.99 (0.82-1.18)	0.94 (0.77-1.14)	0.74*** (0.61-0.89)	0.80* (0.66-0.97)
Maghreb	9,6	1.73*** (1.58-1.90)	1.71*** (1.56-1.88)	1.43*** (1.29-1.59)	1.10 (0.99-1.21)	1.12* (1.01-1.25)
Maghreb naturalized Belgian	7,3	1.32*** (1.18-1.47)	1.32*** (1.18-1.48)	1.25*** (1.11-1.41)	0.94 (0.84-1.05)	1.02 (0.91-1.15)
Sub-Saharan Africa	11,0	1.99*** (1.77-2.24)	1.98*** (1.76-2.23)	1.34*** (1.15-1.55)	1.22** (1.08-1.38)	1.05 (0.91-1.23)
Sub-Saharan Africa naturalized Belgian	8,6	1.56*** (1.28-1.90)	1.57*** (1.29-1.92)	1.42** (1.14-1.77)	1.18 (0.96-1.44)	1.25 (1.00-1.56)
Eastern Europe	7,4	1.33*** (1.12-1.59)	1.30** (1.09-1.56)	1.00 (0.81-1.23)	0.68*** (0.57-0.82)	0.67*** (0.54-0.84)
Eastern Europe naturalized Belgian	4,1	0.73 (0.48-1.11)	0.70 (0.46-1.08)	0.66 (0.41-1.05)	0.52** (0.34-0.79)	0.54* (0.34-0.87)

* ≤ 0.05 ** ≤ 0.01*** ≤ 0.001

^a^adjusted for maternal age and parity

^b^adjusted for maternal age, parity and parental employment status

^c^adjusted for maternal age, parity and maternal education

^d^adjusted for maternal age, parity, parental employment status and maternal education

### Effect of the SES on the association between maternal nationality and perinatal outcomes

In order to study whether the SES is an effect modifier of the association between nationality and perinatal outcomes, we have constructed our models stratified by level of instruction (less than primary versus superior level) and parental employment status (no parents employed versus 2 parents employed) (Table 4  and Table 5). In Table 4, we observe that among households where none of the parents are employed, the rates of LBW for women of all nationalities are significantly lower than for Belgians (*p* < 0.0001). With two parents employed, the picture changes with sub-Saharan African mothers having a significant excess of LBW, whereas Turkish and Eastern European mothers have similar rates to Belgians. Women from EU27 and Maghreb, on the other hand, remain protected against LBW. When analysing maternal education, we observe a similar pattern: with a superior level of education, sub-Saharan African and Turkish mothers have a significant excess of LBW compared to Belgians. Among women with a very low level of education all migrant groups were significantly protected against LBW compared to Belgians.

**Table 4:** Odds Ratios of LBW according to maternal nationality, stratified by parental employment status and maternal education

	cases = 12,911; *n* = 159,978	cases = 33,373; *n* = 769,912	cases = 3,125; *n* = 50,265	cases = 17,239; *n* = 461,142
	0 parents employed# aOR^a^ (95 % CI)	2 parents employed# aOR^a^ (95 % CI)	<primary# aOR^b^ (95 % CI)	Superior# aOR^b^ (95 % CI)
EU27	0.75*** (0.70-0.80)	0.91*** (0.87-0.96)	0.57*** (0.50-0.65)	0.89*** (0.83-0.95)
EU 27 naturalized Belgian	0.82*** (0.73-0.92)	1.03 (0.96-1.10)	0.58*** (0.42-0.79)	1.05 (0.95-1.15)
Turkey	0.50*** (0.44-0.56)	1.02 (0.83-1.26)	0.38*** (0.32-0.44)	1.44** (1.09-1.90)
Turkey naturalized Belgian	0.49*** (0.43-0.56)	0.99 (0.86-1.13)	0.44*** (0.37-0.53)	1.26* (0.99-1.60)
Maghreb	0.34*** (0.31-0.37)	0.73*** (0.63-0.86)	0.27*** (0.24-0.31)	0.75** (0.62-0.90)
Maghreb naturalized Belgian	0.37*** (0.34-0.41)	0.84*** (0.76-0.93)	0.29*** (0.25-0.34)	1.00 (0.88-1.14)
Sub-Saharan Africa	0.59*** (0.55-0.64)	1.52*** (1.27-1.82)	0.50*** (0.44-0.58)	1.26** (1.08-1.47)
Sub-Saharan Africa naturalized Belgian	0.55*** (0.47-0.63)	1.25*** (1.06-1.46)	0.57*** (0.45-0.73)	1.27** (1.07-1.52)
Eastern Europe	0.46*** (0.41-0.51)	0.84 (0.63-1.11)	0.41*** (0.34-0.48)	0.81 (0.63-1.03)
Eastern Europe naturalized Belgian	0.47*** (0.36-0.61)	0.77 (0.59-1.01)	0.48*** (0.34-0.68)	0.79 (0.58-1.08)

The reference group are women with Belgian nationality at delivery and of origin (OR = 1) * ≤ 0.05 ** ≤ 0.01*** ≤ 0.001

^a^Adjusted for maternal age, parity and maternal education

^b^Adjusted for maternal age, parity and parental employment status

The other categories have been omitted for clarity

# Interaction tests are significant for all nationalities (*p* < 0.05)

**Table 5:** Odds Ratios of perinatal mortality according to maternal nationality, stratified by parental employment status and maternal education

	cases = 1,346; *n* = 161,645	cases = 3,021; *n* = 774,096	cases = 373; *n* = 50,798	cases = 1,476; *n* = 463,756
	0 parents employed aOR^a^ (95 % CI)	2 parents employed aOR^a^ (95 % CI)	<primary aOR^b^ (95 % CI)	superior aOR^b^ (95 % CI)
EU27	0.80* (0.65-0.99)#	1.26** (1.09-1.46)#	0.86 (0.56-1.32)	1.15 (0.93-1.44)
EU 27 naturalized Belgian	0.39*** (0.23-0.66)#	0.87 (0.68-1.11)#	0.57 (0.18-1.80)	0.81 (0.56-1.18)
Turkey	1.00 (0.76-1.31)	1.04 (0.57-1.90)	0.95 (0.64-1.41)	2.29** (1.22-4.32)
Turkey naturalized Belgian	0.63* (0.43-0.92)	1.01 (0.67-1.52)	0.61 (0.34-1.09)	1.32 (0.66-2.66)
Maghreb	0.84 (0.69-1.01)	1.44* (1.01-2.06)	1.00 (0.74-1.35)	1.58* (1.04-2.39)
Maghreb naturalized Belgian	0.84 (0.67-1.05)	1.21 (0.93-1.57)	0.90 (0.62-1.30)	1.39 (0.96-2.01)
Sub-Saharan Africa	0.84 (0.69-1.02)#	1.76* (1.06-2.94)#	1.00 (0.66-1.51)	1.41 (0.88-2.26)
Sub-Saharan Africa naturalized Belgian	0.88 (0.60-1.28)	1.06 (0.62-1.84)	1.53 (0.85-2.74)	1.97** (1.21-3.20)
Eastern Europe	0.62*** (0.48-0.81)	0.46 (0.15-1.44)	0.91 (0.57-1.45)	0.37 (0.12-1.16)
Eastern Europe naturalized Belgian	0.35* (0.13-0.94)	0.76 (0.31-1.83)	0.86 (0.32-2.35)	0.73 (0.23-2.27)

The reference group are women with Belgian nationality at delivery and of origin (OR = 1) * ≤ 0.05 ** ≤ 0.01*** ≤ 0.001

^a^Adjusted for maternal age, parity and maternal education

^b^Adjusted for maternal age, parity and parental employment status

The other categories have been omitted for clarity

#Interaction test (*p* < 0.05)

For perinatal mortality (Table 5), a pattern similar to that of LBW is observed, although less pronounced. With none of the parents employed, the rates of perinatal mortality for women of all nationalities (and particularly EU27 and Eastern Europe) are lower than for Belgian women. With two employed parents, the pattern changes with women from Maghreb, sub-Saharan Africa and EU27 having a significant excess of perinatal mortality. Among women with the lowest level of education the rates of perinatal mortality are not significantly different to Belgian mothers. In contrast, with a superior level of education, Maghrebi, sub-Saharan African and Turkish mothers have a significant excess of perinatal mortality compared to Belgian mothers. Again, the acquisition of the Belgian nationality significantly decreased the mortality odds for all the nationalities.

## Discussion

To our knowledge, this is the first study on perinatal health using complete national data in Belgium and exploring the effects of nationality on two perinatal outcomes (LBW and perinatal mortality) taking into account two SES indicators (maternal education and parental employment status). Our research has three main findings: 1) after adjustment for SES, immigrants do not have an excess risk of perinatal mortality and are protected against LBW; 2) immigrant groups are protected against LBW and perinatal mortality among women with low SES but not among those with high SES; 3) immigrant mothers with the Belgian nationality do not have an excess risk of perinatal mortality and maintain their protection against LBW.

### Association between birth outcomes and migration: an outcome and migration-specific process

The association between migration, socioeconomic factors and birth outcomes is not uniform. This association varies notably depending on the considered SES indicator, on the maternal nationality and on the particular outcome. Our results suggest the presence of an outcome and migration-specific process in the way that: risk factors of LBW and perinatal mortality are different and vary depending of the nationality considered.

For instance, regarding the SES, maternal education and parental employment status exert different effects on perinatal outcomes depending on the immigrant group. Adjusting for maternal education decreases the risk of LBW and perinatal mortality as observed in the literature, [16, 17] but in our study, parental employment status seems to weigh more than maternal education for sub-Saharan African mothers. We need further research to explore this observation but this suggests that appropriate measurements of socioeconomic status depend on the population being studied. For instance, the quality of education—and the relative differences in opportunities it represents—may vary across groups [11]. Different measures may capture different aspects of relative or absolute socioeconomic advantage, which may vary in their importance among immigrant groups [11].

Some risk factors for adverse perinatal outcomes appear to be specific to certain migrant sub-groups. For instance, we observe that sub-Saharan African mothers have higher risks of LBW whereas other migrant groups have lower risks of LBW. Various studies have shown that LBW among Sub-Saharan African women is associated to a higher incidence of hypertension and genito-urinary infectionswhereas perinatal mortality among Maghrebi and Turkish women is associated to a higher prevalence foetal or newborn deaths caused by congenital anomalies [12, 18–20].

Our observation are consistent with Urquia et al., who show that the healthy migrant effect is outcome-specific in the way that migrants are healthier with regards to preterm birth, hospitalisation and illness during pregnancy but not with respect to postpartum depression. Moreover, this effect is also ethnicity-specific, since it only applies to non-European immigrants [21]. Similarly, Gould et al. observed that factors conferring protection against LBW in the white population were not protective among Asian Indian population in the US [6].

### Interaction effect of maternal education and parental employment status

A key finding of our study is that being an immigrant is more protective against LBW and perinatal mortality for women with lower SES than for women with higher SES. This confirms previous North-American studies where native-born mothers with a low education had a higher likelihood of adverse birth outcomes compared to foreign-born mothers with a similar education [22, 23]. In addition to the ‘epidemiological paradox’, another paradox has emerged from studies that describe a weak or flat social gradient in health among immigrants [7, 9, 24, 25], which could explain our results. Different hypotheses have been postulated regarding this absence of gradient: it may be due to the fact that health inequalities in the migrants’ countries of origin don’t follow the same social patterns as in high-income countries [24]. Cultural and social factors may be responsible for attenuating the gradients. Indeed, it has been shown that recent migrants tend to have protective health behaviours such as healthier diets, lower consumption of cigarettes and alcohol, and better social support with stronger family ties and social networks [21, 26]. A further possible explanation is related to the ‘healthy migrant effect’ whereby a minimum level of health is thought to be needed to migrate, meaning that only a ‘selection’ of healthy migrants arrive in the host country. The poorer and less healthy people might therefore not be represented, which leads to flattening of the social gradient in health.

It also needs to be considered that the SES indicators which we used in our study may not have the same significance for the Belgian population as for the immigrants. In fact, we tend to assume that mothers with the lowest educational level are at the bottom of the social hierarchy. However, in their countries of origin, women with this educational level may not have been at the bottom of the social ladder. Moreover, the same number of years in school between Belgian and migrant doesn’t necessarily give the same education or the same chances on labor market for migrant population. Additional measures of SES such as occupational status, health insurance, living conditions, social integration, and neighbourhood characteristics may provide better markers of socioeconomic differentiation among immigrants. It has been shown that the effects of area-level socioeconomic characteristics (such as housing quality, safety, and social cohesion) may have an important impact on birth outcomes [11].

### Effect of adopting the Belgian nationality

In our study, we observe a favourable effect of adopting the Belgian nationality on birth outcomes, which replicates our previous findings from Brussels [12]. However, this effect is less pronounced for LBW compared with perinatal mortality. In Belgium the nationality can be acquired in three ways: by statement, marriage or naturalization. The statement and marriage are open to adults who meet a series of requirements (related length of residence, place of birth, attachments to Belgium). For naturalization, a principal residence, covered by a legal residence in Belgium for at least three years (2 years for refugees) is required. The procedure takes at least 18 months and does not depend on cultural or language knowledge, nor on medical conditions [27]. The current nationality of the mother is a good way to assess the effects of first generation migration whereas the effects of 2nd or 3rd generation can be better approached by the use of the nationality of origin.

It has been shown that birth outcomes may either improve or deteriorate with length of residence, depending on the migrant group [28]. A common framework for the interpretation of these patterns is provided by the ‘acculturation theory’, which describes a socio-cultural process in which members of one cultural group adopt the beliefs and behaviours of another group [29, 30]. For example, the public health literature suggests that although immigrants from Latin America may arrive in the US with relatively healthy behaviours, they gradually disappear during the process of acculturation with higher proportions of preterm birth and morbidity during pregnancy [31]. In our study, we might view the acquisition of the Belgian nationality as a proxy indicator of acculturation, which exerts, in contrast, a protective effect. LBW did not increase and perinatal mortality decreased among women who acquired the Belgian nationality. These findings are consistent with Cacciani et al.’s, who found that perinatal outcomes improved over time in some immigrant women [32]. The acquisition of the Belgian nationality is more likely to be granted to people who have a longer duration of stay in Belgium, higher education, legal status and better language skills; which is indirectly linked to better access to quality health care, a key determinant to reduce adverse perinatal outcomes [33, 34].

## Limitations

This study has some limitations. We ought to recognise that measuring acculturation through the acquisition of the Belgian nationality bears its own limitations, as it doesn’t directly measure aspects such as length of stay in the country, generation status, French or Dutch language skills, and other social and cultural factors, and it may depend on administrative procedures. Further research is needed to take these elements into account and to analyze the association of perinatal outcomes with the length of stay in the country.

Another limitation concerns the high proportion of maternal educational levels classified as ‘other’. In our national database we can not have details into what the “other” group includes. However, in previous studies, our Brussels database has shown that the education level includes a lot of missing values derived from death certificates [13]. We suppose that workers at the civil registration office feel uneasy about asking questions regarding the parents’ level of education when they are declaring the death of their child. We lacked data concerning additional measures of SES. Further research exploring longitudinally the socio-economic indicators among immigrants would be helpful.

We also lacked information about maternal health, antenatal care, cultural practices and health behaviours (such as diet, smoking, alcohol and drug consumption) which prevents us from exploring their impact on the perinatal outcomes. Particularly, a growing field of research have shown that adverse perinatal outcomes among immigrants are associated with late or inadequate prenatal and obstetric care due to the difficulty in accessing services [35] and with stressful live events [36].

## Conclusions

In conclusion, our study contributes to a better understanding of how socioeconomic factors interact with the association between perinatal outcomes and immigration in Belgium. It reveals a protective effect of migration on perinatal outcomes among women with low SES, and underlines the importance of taking into account the socioeconomic status in order to more fully understand the relationship between migration and perinatal health. We need further studies to better measure the socioeconomic characteristics among immigrants, such as longitudinal measurements on migration, including pre-migration characteristics, social support, neighbourhood characteristics, access to and use of medical care, SES indicators and health behaviours.
